# C5aR1 Promotes Invasion, Metastasis, and Poor Prognosis in Cutaneous Squamous Cell Carcinoma

**DOI:** 10.1016/j.ajpath.2025.02.004

**Published:** 2025-03-06

**Authors:** Lauri Heiskanen, Liisa Nissinen, Elina Siljamäki, Jaakko S. Knuutila, Teijo Pellinen, Markku Kallajoki, Jyrki Heino, Pilvi Riihilä, Veli-Matti Kähäri

**Affiliations:** ∗ Department of Dermatology, University of Turku and Turku University Hospital, Turku, Finland; † FICAN West Cancer Research Laboratory, University of Turku and Turku University Hospital, Turku, Finland; ‡ Department of Life Technologies and InFLAMES Research Flagship, University of Turku, Turku, Finland; § MediCity Research Laboratory, University of Turku, Turku, Finland; ¶ Institute for Molecular Medicine Finland (FIMM), Helsinki Institute of Life Science (HiLIFE), University of Helsinki, Helsinki, Finland; ‖ Department of Pathology, University of Turku and Turku University Hospital, Turku, Finland

## Abstract

Cutaneous squamous cell carcinoma (cSCC) is the most common metastatic skin cancer, and the metastatic from is associated with a poor prognosis. Here, the role of the complement C5a receptor C5aR1 was examined in the progression and metastasis of cSCC. C5aR1 expression was increased in cSCC cells in a three-dimensional spheroid coculture model in the presence of fibroblasts, and treatment with recombinant C5a enhanced the invasion of cSCC cells. Staining for C5aR1 was detected on the surface of tumor cells at the invasive edge of human cSCC xenografts *in vivo*. Metastatic and non-metastatic primary human cSCCs, premalignant and benign epidermal lesions, and normal skin for C5aR1 were stained with multiplex immunofluorescence and chromogenic immunohistochemistry. Increased expression of C5aR1 was observed on the surface of tumor cells and fibroblasts in invasive cSCCs and recessive dystrophic epidermolysis bullosa–associated cSCCs compared with cSCC *in situ*, actinic keratoses, seborrheic keratoses, and normal skin. Increased expression of C5aR1 on the tumor cell surface and in fibroblasts was associated with metastatic risk and poor disease-specific survival of patients with primary cSCC. These findings suggest a role of C5a in cSCC cell invasion, and they identify C5aR1 as a novel biomarker for metastasis risk and poor prognosis in patients with cSCC. The results also suggest that C5aR1 could be a novel therapeutic target for the treatment of locally advanced and metastatic cSCC.

## Graphical abstract

**Figure:**
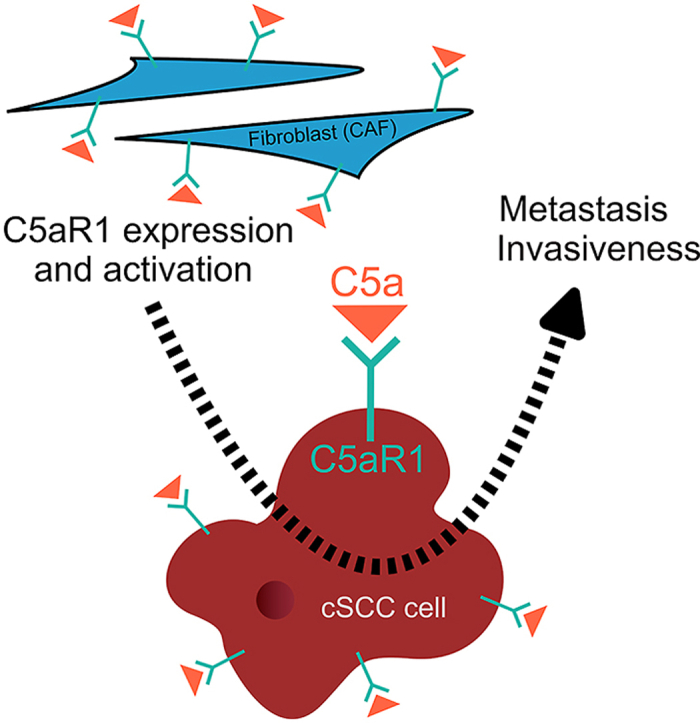

Cutaneous squamous cell carcinoma (cSCC) is the most common metastatic skin cancer, with an increasing incidence in recent decades.^1^, ^2^, ^3^ Exposure to solar UV radiation is the predominant risk factor for cSCC, which commonly arises from the precursor lesions actinic keratosis (AK) and Bowen’s disease [*in situ* cSCC (cSCCIS)] in sun-damaged skin.^4^ Approximately 3% to 5% of primary cSCCs metastasize, and the prognosis for patients with metastatic cSCC (mcSCC) is poor.^5^^,^^6^ cSCC accounts for nearly 25% of annual skin cancer deaths.^7^ Currently, there are no established molecular markers in clinical practice for predicting the metastasis risk of primary cSCCs. Therefore, there is a need for predictive biomarkers for the prognosis of cSCC and for new therapeutic targets for mcSCC.

The complement system is an integral part of human innate immunity, and its role in cancer progression has recently been emphasized.^8^, ^9^, ^10^ Tumor cell–specific expression of several complement components (FB, FD, C3, C1r, and C1s) and inhibitors (FH and FI) has been documented in cSCC, and they play a non-canonical role in cSCC progression *in vivo*.^11^, ^12^, ^13^, ^14^, ^15^, ^16^, ^17^ The complement component C5a functions as a vasodilator and chemotactic factor, and it increases vascular permeability and degranulation of mast cells by attaching to the specific receptor C5aR1 on the target cells.^18^, ^19^, ^20^ C5aR1 is up-regulated in infectious and inflammatory diseases such as sepsis, respiratory distress syndrome, systemic lupus erythematosus, and inflammatory bowel disease.^21^, ^22^, ^23^ C5aR1 is overexpressed in several cancer types, including non–small cell lung cancer, urothelial cell carcinoma, renal cell carcinoma, gastric cancer, hepatocellular carcinoma, prostate cancer, and breast cancer,^24^, ^25^, ^26^, ^27^, ^28^, ^29^, ^30^, ^31^, ^32^ and is associated with poor survival.^33^ In addition, C5a production is up-regulated in multiple cancers, and its presence is linked to increased metastatic potential of cancer cells.^34^ C5a can also modulate the immune microenvironment toward a pro-tumor or antitumor response depending on the tumor type and local concentration of C5a.^35^

The expression of C5aR1 mRNA has not been detected in cSCC cells or normal keratinocytes cultured in a monolayer.^12^ The aim of the current study was to further examine the role of C5aR1 in the progression and metastasis of cSCC. The results show increased expression of C5aR1 in cSCC cells cocultured with human skin fibroblasts in three-dimensional (3D) spheroids and increased invasion of cSCC cells through collagen upon treatment with recombinant C5a. Elevated expression of C5aR1 was noted in the cSCC tumor cells and stromal fibroblasts compared with AK and cSCCIS *in vivo*, with the expression increasing toward mcSCC and cSCC metastases. Moreover, the up-regulation of C5aR1 in cSCC cells and fibroblasts in the tumor microenvironment (TME) was associated with poor prognosis. These findings suggest that C5aR1 may serve as a prognostic biomarker and a therapeutic target for locally advanced and mcSCC.

## Materials and Methods

### Ethical Issues

The study was approved by the Ethics Committee of the Hospital District of Southwest Finland (187/2006) and the Scientific Steering Committee (AB15-9721) of Auria Biobank (Turku, Finland). The research was performed according to the Declaration of Helsinki, and an informed biobank consent was obtained from the patients. Registry study approval for collection and use of clinical and histopathologic data was obtained from the Turku University Hospital Clinical Research Centre (TO5/042/18; Turku, Finland). All experiments with mice were conducted with permission of the Animal Test Review Board of Southern Finland (ESAVI15107/2020) according to institutional guidelines.

### Cell Lines

Primary non-metastatic (UT-SCC-91) and metastatic (UT-SCC-7 and UT-SCC-115) cell lines were established from surgically removed cSCCs at Turku University Hospital.^36^^,^^37^ The authentication of these cell lines was performed by STR DNA profiling (DDC Medical, Fairfield, OH).^36^ The Ha-*ras*-transformed tumorigenic HaCaT cell line RT3^38^ was kindly provided by Dr. Norbert Fusenig (German Cancer Research Center, Heidelberg, Germany). Primary adult human skin fibroblasts from a 24-year–old male donor were a kind gift from Prof. Risto Penttinen (University of Turku, Turku, Finland).^39^^,^^40^ Normal human adult dermal fibroblasts (C-12302) were purchased from PromoCell GmbH (Heidelberg, Germany). Both fibroblast strains were used up to passage number 12. All cell lines were grown in Dulbecco’s modified Eagle’s medium [(DMEM) with 4.5 g/L glucose; 12-614F; Lonza, Verviers, Belgium] supplemented with 10% fetal calf serum, l-glutamine (6 nmol/L), penicillin (100 U/mL), and streptomycin (100 μg/mL). Then, 1 × MEM non-essential amino acids (11140-035; Gibco, Carlsbad, CA) were added to the medium of cSCC cell lines. Geneticin-418 (200 μg/mL; 10131035; Gibco) was added to the RT3 cell line medium. The cell lines were routinely tested to be negative for mycoplasma contamination by using a MycoAlert PLUS Mycoplasma Detection Kit (LT07-710; Lonza, Cologne, Germany).

### 3D Spheroid Cultures

The information on experimental parameters, spheroid preparation, and growth conditions of spheroids is based on MISpheroID guidelines and recommendations.^41^ For Western blot analysis and invasion assays, 3D spheroids were made in micro-molds according to the manufacturer’s instructions (MicroTissues 3D Petri Dish micro-mold spheroids; Sigma-Aldrich, St. Louis, MO) with 2.5 × 10^5^ cells in one mold (monocultures; 7000 cells per spheroid) or 5.0 × 10^5^ cells in one mold (cocultures; 14,000 cells per spheroid). In cocultures, the cell ratio was 1:1 (RT3*/*UT-SCC-7/UT-SCC-115 cells and fibroblasts, respectively). The spheroids were grown in serum-free DMEM for 3 days at 37°C in an incubator environment of 20% oxygen and 5% carbon dioxide. Ascorbic acid (50 μg/mL) was added daily.

### CellTracker Labeling

RT3, UT-SCC-7, and UT-SCC-115 cell lines and primary human skin fibroblasts were labeled with CellTrackers in two-dimensional conditions: RT3, UT-SCC-7, and UT-SCC-115 cells in red (CellTracker Orange CMTMR Dye, C2927; Invitrogen, Waltham, MA) and fibroblasts in green (CellTracker Green CMFDA Dye, C2925; Invitrogen). The cells were labeled with 2.5 μM dye in DMEM supplemented with 10% fetal calf serum for 1 hour at 37°C, washed twice with phosphate-buffered saline (PBS), and constructed into spheroids. After 3 days, the spheroids were used in invasion assays or fixed for immunofluorescence staining.

### Immunofluorescence Staining of Spheroids

Three-day–old spheroids were fixed with 4% paraformaldehyde for 1 hour at 4°C, followed by fixation with 4% paraformaldehyde supplemented with 1% Triton X-100 for 1 hour at 4°C. The spheroids were washed twice with PBS + 0.1% Triton X-100 (PBST) and blocked with 6% bovine serum albumin (BSA) in PBST for 3 hours at room temperature (20°C-25°C). C5aR1 antibody (1:100 in PBST; ab234757; Abcam, Cambridge, UK) was incubated for 3 hours at room temperature, and spheroids were then washed three times with PBST (15 minutes each wash). The spheroids were then treated with highly pre-cross absorbed Alexa Fluor 633 goat anti-rabbit IgG secondary antibody (1:200 in PBST; A21071; Invitrogen, Carlsbad, CA) for 1.5 hours at room temperature and washed three times with PBST (15 minutes each wash). The spheroids were mounted in 95% glycerol.

### Invasion Assays

For invasion assays in 3D spheroids in collagen I, the cells were stained with CellTrackers as described in CellTracker Labeling, constructed into spheroids, and allowed to grow for 3 days. Ascorbic acid (50 μg/mL in serum-free DMEM medium) was added daily. Three-day–old spheroids were plated on collagen I–coated 96-well plates (0.035 mg/mL; collagen solution from bovine skin; C4243; Sigma-Aldrich), and collagen I gel (2.0 mg/mL; Type I Bovine Collagen Solution, #5010; Nutragen, Advanced BioMatrix, Carlsbad, CA) was layered on top of the spheroids. DMEM supplemented with 10% fetal calf serum was added above the collagen gel. To study the effect of C5a on cell invasion, 3-day–old spheroids were incubated with 100 nmol/L recombinant human complement component C5a (rhC5a) (#2037-C5; R&D Systems, Minneapolis, MN) for 3 hours at 37°C before embedding the spheroids into the collagen gel. Control samples were incubated with 0.1% BSA-PBS for 3 hours at 37°C. The collagen I gel and the medium on top of the collagen I gel were supplemented with 100 nmol/L rhC5a and in control samples with 0.1% BSA-PBS.

To exclude the effect of proliferation on cell invasion, 3-day–old spheroids were incubated with 1 mmol/L hydroxyurea (HU) (H8627; Sigma-Aldrich) for 3 hours at 37°C before embedding the spheroids into the collagen gel. Control samples were incubated with 0.1% BSA-PBS for 3 hours at 37°C. The medium on top of the collagen I gel was supplemented with 0.5 mmol/L HU and in control samples with 0.1% BSA-PBS. Spheroids were allowed to invade for 96 hours and were imaged every 24 hours with an LSM 880 confocal microscope (Zeiss, Jena, Germany). Fiji (*https://Fiji.sc*), an image processing platform based on ImageJ version 2.0.0-rc-69/1.52n^42^ (NIH, Bethesda, MD; *https://imagej.net/ij*), was used to calculate the invasion (ie, the area covered by cells). The cell invasion of two to six spheroids from each sample were analyzed. Biological replicates are stated in the figure legends.

For invasion assays in inserts, collagen gel was prepared by mixing type I collagen (PureCol; Advanced BioMatrix, San Diego, CA) with 5 × DMEM and 0.2 mol/L HEPES buffer (pH 7.4) at a ratio of 7:2:1, respectively. Sodium hydroxide (1 mol/L) was added to obtain a final pH of 7.4. After labeling of UT-SCC-7 cells and fibroblasts with CellTrackers, the cells were trypsinized, suspended in DMEM containing 0.1% BSA, and seeded (both cell types 2.5 × 10^5^ cells per insert) to collagen I–coated invasion chambers. The cell suspension was supplemented with 100 nmol/L rhC5a and in control samples with 0.1% BSA-PBS. Chemoattractant (10% fetal bovine serum in DMEM) was added into the lower chamber supplemented with 100 nmol/L rhC5a and in control samples with 0.1% BSA-PBS. After 48 hours’ incubation, cells on the upper surface of the insert were removed, and the invaded cells on the lower surface were fixed with methanol. UT-SCC-7 cells and fibroblasts were then counted under a fluorescent microscope.

### Confocal Imaging

The spheroids were imaged with an LSM 880 Airyscan confocal microscope (Zeiss) (10× and 20× objectives; green CellTracker excitation at 488 nm, orange CellTracker excitation at 543 nm, and Alexa Fluor 633-secondary antibody excitation at 633 nm). The imaging was performed at the Cell Imaging and Cytometry Core, Turku Bioscience Centre (Turku, Finland), with the support of Biocenter Finland.

### Gene Expression Profiling Interactive Analysis

The online Gene Expression Profiling Interactive Analysis (GEPIA, *http://gepia.cancer-pku.cn*, last accessed December 1, 2024) analysis tool was used to analyze the relationship between C5AR1 mRNA expression and prognosis of esophageal carcinoma and lung SCC in The Cancer Genome Atlas data.^43^^,^^44^

### Human cSCC Xenografts

Human cSCC xenografts were established as previously described.^13^ Primary UT-SCC-91 (7 × 10^6^) and metastatic UT-SCC-7 cells (5 × 10^6^) were injected subcutaneously into the back of 6-week–old severe combined immunodeficient mice (CB17/Icr-Prkdc^scid^/IcrIcoCrl) (Charles River Laboratories, Minneapolis, MN). UT-SCC-91 and UT-SCC-7 xenograft tumors were harvested after 16 or 21 days, respectively, and processed for immunohistochemical (IHC) analysis as described previously.^13^

### Tissue Material and Chromogenic Immunohistochemistry

Tissue samples were collected from Auria Biobank and the archives of Turku University Hospital. Tissue microarrays consisting of formalin-fixed, paraffin-embedded human tissue specimens obtained by resection or biopsy were constructed, and the characteristics of tumor cohorts were previously described (Supplemental Table S1).^5^^,^^45^ The tissue samples consisted of normal skin (*n* = 54 individual skin samples), seborrheic keratosis (SK; *n* = 6 individual tumors), AK (*n* = 50 individual tumors), cSCCIS (*n* = 51 individual tumors), non-mcSCC (*n* = 152 samples, 97 individual tumors), mcSCC (*n* = 77 samples, 55 individual tumors), cSCC metastases (*n* = 94 samples, 65 individual tumors), and recessive dystrophic epidermolysis bullosa–associated cSCC (RDEBSCC; *n* = 11 individual tumors). The characteristics of the RDEBSCC samples are described in Supplemental Table S2. Tissue microarrays contained replicate cores from each tumor. The findings from tumors with multiple spots were merged to one so that one result per tumor or skin sample was observed.

### Multiplexed Immunofluorescence

C5aR1 and activated fibroblast markers, along with their distribution in tumor tissue, were defined by using multiplexed immunofluorescence (mIF). The first panel contained C5aR1 and fibroblast activation protein (FAP), the second panel CD45 and α-smooth muscle actin (αSMA), and the third round of staining with PanEpi (Table 1).^45^ C5aR1 expression levels were analyzed based on the relative cell area in normal skin samples (*n* = 62 spots, 54 individual skin samples), AKs (*n* = 54 spots, 53 individual tumors), cSCCIS (*n* = 47 spots, 47 individual tumors), invasive cSCCs (*n* = 198 spots, 174 individual tumors), cSCC metastases (*n* = 9 spots, 9 individual tumors), and RDEBSCCs (*n* = 83 spots, 11 individual tumors). When multiple spots were derived from the same tumor, only the highest observed result from all spots was included in the data to capture the hot spot of expression. This approach was chosen based on the rationale that areas of maximum C5aR1 expression may represent the regions of greatest significance, especially with respect to metastatic potential in cSCC.

**Table 1:** Antibodies Used in the Study

Experiment	Antibody/product
Spheroid IF	
A21071	Alexa Fluor 633 goat anti-rabbit IgG secondary antibody (A21071, Invitrogen; 1:200 in PBST)
Ab234757	C5aR1 antibody (ab234757, Abcam; 1:100 in PBST)
Xenograft IHC	
Ab11867	C5aR1-antibody [C5a-R (S5/1) (Abcam/ab11867)]
Multiplexed IF	
TSA-555	Ra FAP (ab207178) 1:500
Alexa Fluor 647	Ma C5aR1 (ab11867) 1:100
Alexa Fluor 750	Ma SMA (DAKO M0851) 1:200; O/N 4°C
Alexa Fluor 647	R-anti-CD45 (CST13917) 1:100
Alexa Fluor 750	PanEpi
Chromogenic IHC	
Ab11867	C5aR1-antibody [C5a-R (S5/1) (Abcam/ab11867)]

FAP, fibroblast activation protein; IF, immunofluorescence; IHC, immunohistochemistry; PBST, phosphate-buffered saline + 0.1% Triton X-100; SMA, smooth muscle actin.

### Statistical Analysis

All quantitative data regarding coculture models are presented as means ± SD or SEM as stated in the figure legends. The Shapiro-Wilk test was used to test normality assumption. The Levene test was used to test the homogeneity of variances between the statistically compared groups. Statistical differences were determined by using either paired *t*-test or analysis of variance complemented by appropriate post hoc tests [Tukey (if variances between the statistically compared groups were similar) or Dunnett’s T3 (if variances between statistically compared groups were not similar)]. Origin 2015 (OriginLab Corporation, Northampton, MA) and SPSS version 25 (IBM Corp., Armonk, NY) were used to perform the analyses. Only two-tailed *P* values < 0.05 were considered as statistically significant.

Statistical analysis of IHC staining and survival analysis were made with JMP version Pro 16 (SAS Institute, Inc., Cary, NC; 1989-2024). The semiquantitative analysis included four classes based on C5aR1 staining rate: negative staining (–), weak staining (+), moderate staining (++), and strong staining (+++). For statistical analysis, these classes were grouped in two classes, weak staining including (–) and (+) groups, and strong staining including (++) and (+++) groups. The statistical analysis of semiquantitative data was made with the Fisher exact test. Survival analysis was made with Kaplan-Meier curves and log-rank analysis.

For disease-specific survival analysis, 39 patients were identified with weak cSCC cell surface C5aR1 intensity and 37 patients with strong cSCC cell surface C5aR1 intensity. In addition, for the analysis of TME fibroblast C5aR1 intensity, 46 patients with weak C5aR1 intensity and 25 patients with strong C5aR1 intensity were included in our data set. Five of the original histopathologic samples lacked stromal tissue, and fibroblasts were therefore not included in these samples. Only two-tailed *P* values <0.05 were considered as statistically significant. For mIF, the statistical analyses were made with the Kruskal-Wallis test, and comparisons for each pair were implemented with the Steel-Dwass method.

## Results

### C5aR1 Expression in cSCC Cells Is Induced by Co-culture with Fibroblasts

**Figure 1:**
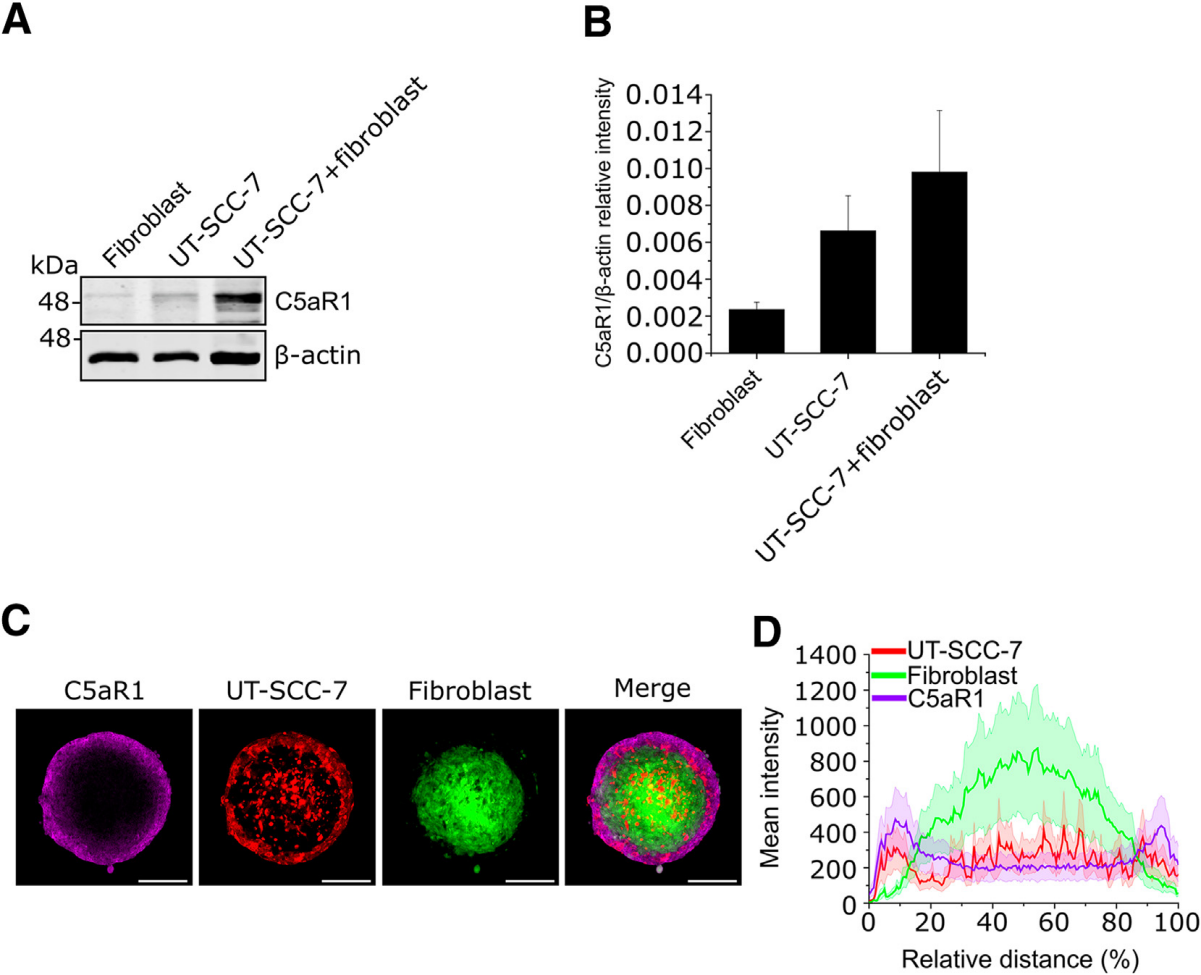
Fibroblasts induce C5aR1 expression in cutaneous squamous cell carcinoma (cSCC) cells. **A:** Western blot analysis of C5aR1 in three-dimensional spheroids composed of human skin fibroblasts, metastastic cSCC cells (UT-SCC-7), and their coculture. A representative image from three independent biological replicates is presented. β-actin was used as a loading control. **B:** Quantification of C5aR1 levels from the Western blot analysis represented in **A**. The graph illustrates C5aR1 relative intensity to β-actin ± SEM. Three independent biological replicates were conducted. **C:** Confocal images of spheroids with cSCC cells (UT-SCC-7) cocultured with human skin fibroblasts. The cells were initially labeled with CellTrackers (UT-SCC-7 cells in red and fibroblasts in green), and the spheroids were allowed to grow for 3 days. After paraformaldehyde fixation, the spheroids were subjected to immunofluorescence staining for C5aR1. Three independent biological replicates were performed. **D:** Expression profile of spheroids containing UT-SCC-7 cells and human skin fibroblasts. The cells were treated as in **C**, and the expression profile was calculated from the confocal images. Mean intensity values depict the intensities of UT-SCC-7 cells (red), fibroblasts (green), and C5aR1 (magenta), while the relative distance indicates the diameter of the spheroids. Twenty-four spheroids from three biological replicates were analyzed. Means (dark line) ± SEM (light area around the line) is shown. *n* = 24 spheroids (**C**). Scale bars: 200 μm (**C**).

**Supplemental Figure S1:**
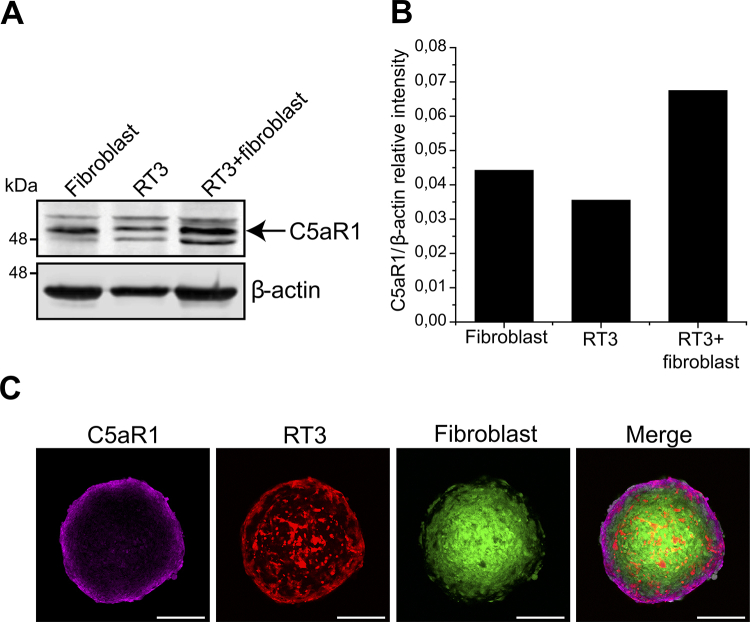
C5aR1 expression in RT3 cells is induced by coculture with fibroblasts. **A:** Western blot analysis of C5aR1 in three-dimensional spheroids composed of human skin fibroblasts, metastastic Ha-*ras*-transformed HaCaT cells (RT3), and their coculture. β-actin was used as a loading control. **B:** Quantification of C5aR1 levels from Western blot analysis in A. The graph illustrates C5aR1 relative intensity to β-actin ± SEM. **C:** Confocal images of spheroids with RT3 cells cocultured with human skin fibroblasts. The cells were initially labeled with CellTrackers (RT3 cells in red and fibroblasts in green), and the spheroids were allowed to grow for 3 days. After paraformaldehyde fixation, the spheroids were subjected to immunofluorescence and stained for C5aR1. *n* = 2 (**A** and **B**); *n* = 2, 16 spheroids imaged (**C**). Scale bars: 200 μm (**C**).

C5aR1 expression is not detected at the mRNA level in cSCC cells cultured in a monolayer.^12^ To further examine the role of C5aR1 in cSCC, cocultures of cSCC cells and normal human skin fibroblasts in a 3D spheroid model were used.^39^^,^^40^ In cocultures of mcSCC cells (UT-SCC-7) and fibroblasts, C5aR1 expression was markedly increased compared with spheroids that contained only cSCC cells or fibroblasts (Figure 1, A and B). Confocal imaging showed peripheral localization of UT-SCC-7 cells, whereas fibroblasts were primarily located in the center of the spheroid (Figure 1C). Immunofluorescent staining revealed co-localization of C5aR1-positive cells with UT-SCC-7 cells in the outer shell of the spheroid (Figure 1C). Confocal imaging results were verified by a computational analysis method that allowed analysis of the expression profiles of the spheroids, taking into account different diameters and the intensity values of the spheroids. The expression profile analysis showed C5aR1 expression emerging from the spheroid edges, where UT-SCC-7 cells were also located (Figure 1D). To verify the results obtained with UT-SCC-7 cells, Ha-*ras*-transformed metastatic epidermal HaCaT keratinocytes (RT3 cells) were cultured together with human skin fibroblasts. Western blot analysis revealed that C5aR1 expression was increased in spheroids containing both RT3 cells and fibroblasts (Supplemental Figure S1, A and B). Confocal imaging showed that C5aR1 expression was most prominent in the outer shell of the spheroids, where RT3 cells were also located (Supplemental Figure S1C).

### C5a Increases cSCC Cell Invasion in Collagen I

**Figure 2:**
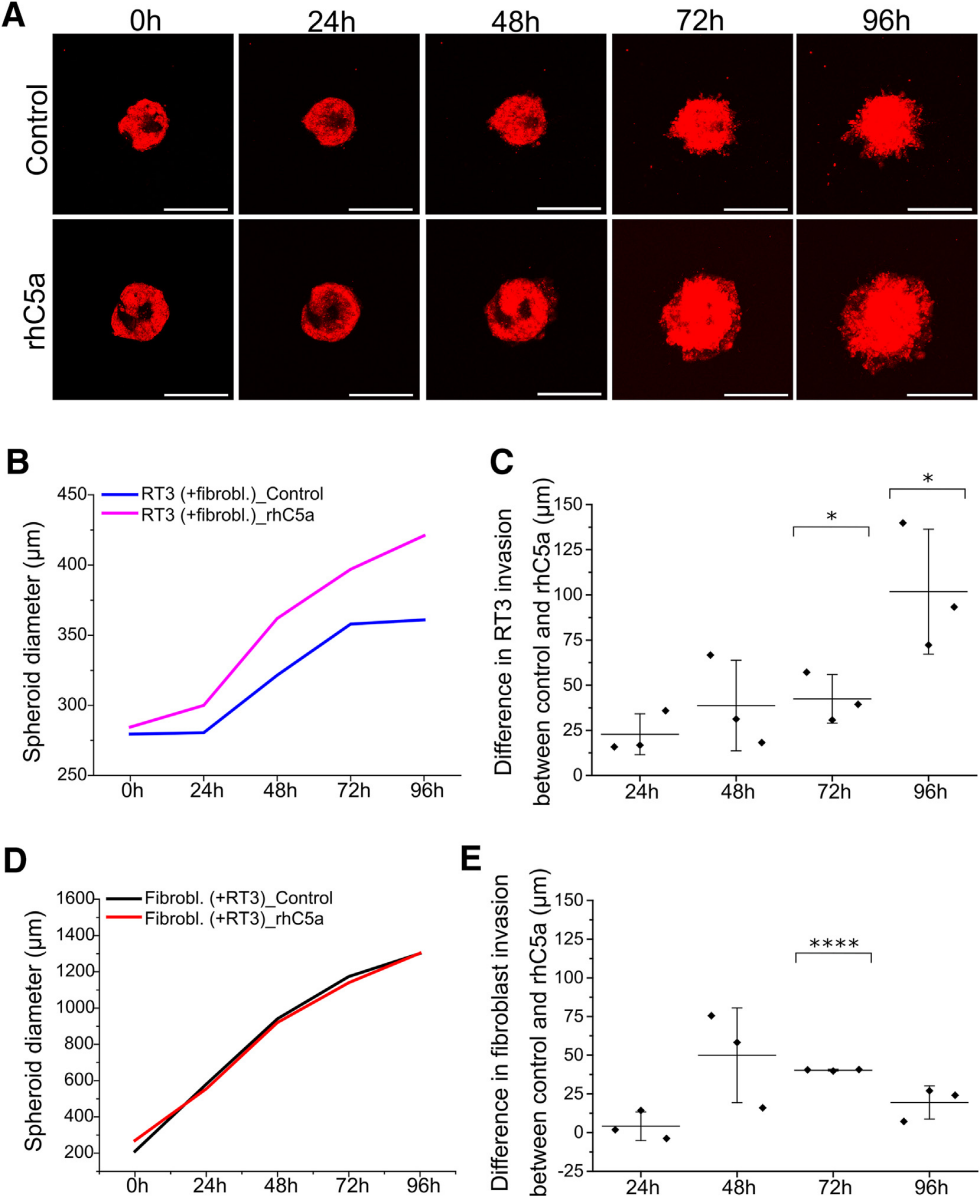
Recombinant human complement component C5a (rhC5a) increases RT3 cell invasion in collagen I. **A:** RT3 cells (red) were incorporated into spheroids with human skin fibroblasts, and the spheroids were allowed to grow for 3 days. The spheroids were then treated with rhC5a for 3 hours, transferred to a 96-well plate, and embedded in a collagen I gel. Invasion was monitored by using a confocal microscope every 24 hours over 5 days. For each time point, two to six spheroids were imaged and analyzed. Three independent biological replicates were performed. **B** and **C:** RT3 cell invasion from cocultured spheroids that were treated as described in **A**. A representative graph from three biological replicates (**B**) and analysis of RT3 cell invasion from cocultured spheroids (**C**). The graph illustrates the difference in RT3 cell invasion between control samples and rhC5a-treated samples. The graph displays the mean from three independent biological replicates (squares) ± SD (each replicate contained two to six spheroids). **D** and **E:** Fibroblast invasion from cocultured spheroids that were treated as described in **A**. A representative graph from three biological replicates (**D**), and analysis of fibroblast invasion from cocultured spheroids (**E**). The graph indicates the difference in fibroblast invasion between control samples and rhC5a-treated samples. The graph displays the mean from three independent biological replicates (squares) ± SD (each replicate contained two to six spheroids). *P* values are from paired *t*-tests. ∗*P* < 0.05; ∗∗∗∗*P* < 0.0001. Scale bars: 400 μm (**A**).

**Supplemental Figure S2:**
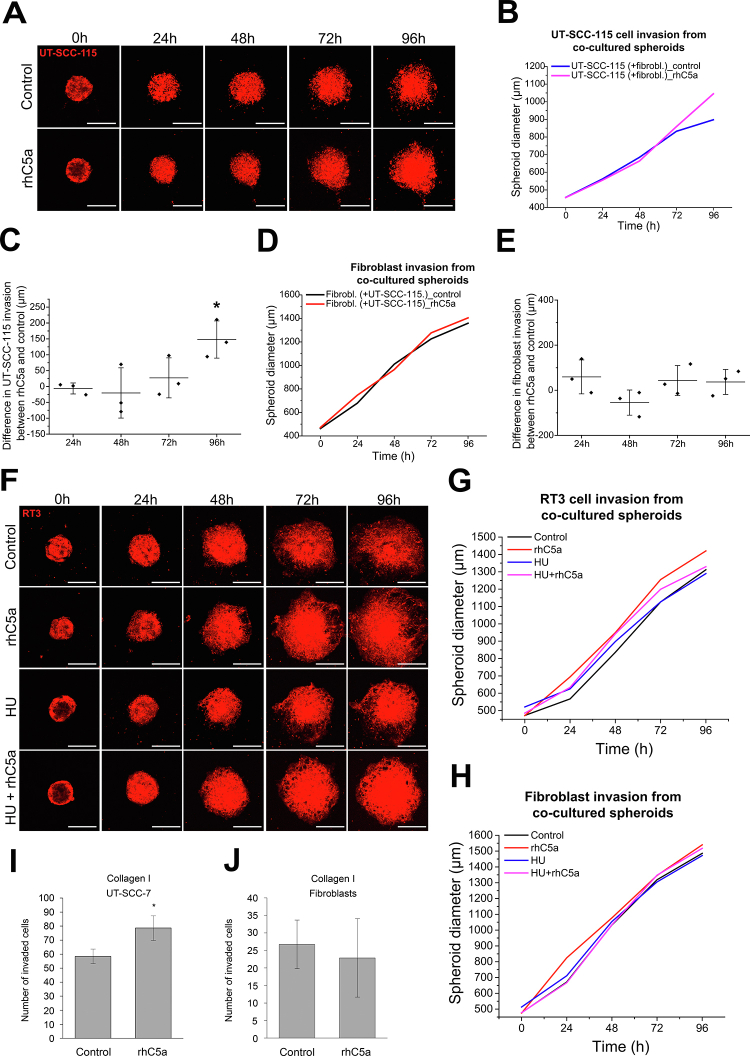
Recombinant C5a increases cell invasion in collagen I. **A:** Metastatic UT-SCC-115 cells (red) were constructed into spheroids with primary human skin fibroblasts, and the spheroids were allowed to grow for 3 days. The spheroids were then treated with recombinant human complement component C5a (rhC5a) (100 nmol/L) for 3 hours, after which they were transferred to a 96-well plate and embedded within a collagen I gel. The invasion was monitored by using a confocal microscope every 24 hours over 5 days. From each time point, two to five spheroids were imaged and analyzed. Three independent biological replicates were performed. **B** and **C:** UT-SCC-115 cell invasion from cocultured spheroids that were treated as in **A**. **B:** Graph from the mean of three biological replicates. **C:** Analysis of UT-SCC-115 cell invasion from cocultured spheroids. The graph shows the difference in UT-SCC-115 cell invasion between control samples and rhC5a-treated samples. It also shows the means from three independent biological replicates (squares) ± SD. Each replicate contained two to five spheroids. The *P* value is from a paired *t*-test. **D** and **E:** Fibroblast invasion from cocultured spheroids that were treated as in **A**. **D:** Graph from the means of three biological replicates. **E:** Analysis of fibroblast invasion from cocultured spheroids. The graph shows the difference in fibroblast invasion between control samples and rhC5a-treated samples. It also shows the mean from three independent biological replicates (squares) ± SD. Each replicate contained two to five spheroids. **F:** RT3 cells (red) were constructed into spheroids with primary human skin fibroblasts, and the spheroids were allowed to grow for 3 days. The spheroids were then treated with either rhC5a (100 nmol/L) for 3 hours, with hydroxyurea (HU, 1 mmol/L) for 3 hours, or with a combination of both rhC5a and HU for 3 hours. Control cells were treated with phosphate-buffered saline (PBS)/0.1% bovine serum albumin (BSA) for 3 hours. The spheroids were then transferred to a 96-well plate and embedded within a collagen I gel. The invasion was monitored by using a confocal microscope every 24 hours over 5 days. From each time point, three to five spheroids were imaged and analyzed. **G:** RT3 cell invasion from cocultured spheroids that were treated as in **F**. **H:** Fibroblast invasion from cocultured spheroids that were treated as in **F**. **I** and **J:** UT-SCC-7 cells (**I**) and fibroblasts (**J**) were labeled with CellTrackers and seeded to the upper chamber of the tissue culture inserts (with 8.0 μm pore size) coated with collagen I. The cell suspension was supplemented with 100 nmol/L rhC5a, and in control samples with 0.1% BSA-PBS. Chemoattractant (10% fetal bovine serum in Dulbecco’s modified Eagle’s medium) was added into the lower chamber supplemented with 100 nmol/L rhC5a, and in control samples with 0.1% BSA-PBS. After 48 hours, invaded cells were fixed and counted. Data are expressed as means ± SD. *n* = 3. Statistical significance was determined by using the *t*-test. ∗*P* < 0.05. Scale bars: 400 μm (**A** and **F**).

To investigate the potential role of C5a in cell invasion, 3D spheroids established with RT3 cells and human skin fibroblasts were treated with rhC5a. Confocal images showed that rhC5a treatment enhanced RT3 cell invasion compared with the control cultures (Figure 2A). Quantitative analysis revealed that treatment with rhC5a significantly increased the invasion of RT3 cells out of spheroids at 72- and 96-hour time points compared with untreated control cells (Figure 2, B and C). The invasion of fibroblasts out of the same spheroids remained unaffected by rhC5a treatment except for the 72-hour time point (Figure 2, D and E). To verify the results obtained with the RT3 cell line, the same invasion assay was performed with the metastatic UT-SCC-115 cell line and human skin fibroblasts. Compared with control cells, treatment with rhC5a increased UT-SCC-115 cell invasion out of the cocultured spheroids in later time points (Supplemental Figure S2, A and B), and the increase was statistically significant at the 96-hour time point (Supplemental Figure S2C). Fibroblast invasion from the same spheroids was unaffected by rhC5a treatment (Supplemental Figure S2, D and E).

To exclude the possibility that cell invasion out of the spheroids was due to cell proliferation, cell proliferation was prevented by treating the spheroids containing RT3 cells and primary human fibroblasts with 1 mmol/L HU 3 hours before the invasion assay. HU (0.5 mmol/L) was also present in the attraction medium throughout the whole invasion assay. Confocal images showed that RT3 cell invasion was not affected by HU treatment because the cells invaded equally well with and without HU (Supplemental Figure S2F). Control cells and HU-treated cells invaded in a similar fashion (Supplemental Figure S2G). In addition, compared with control samples, rhC5a treatment increased RT3 cell invasion out of the spheroids with or without HU treatment (Supplemental Figure S2, F and G). Fibroblast invasion was not affected by HU or rhC5a treatment (Supplemental Figure S2H).

To confirm the effect of rhC5a on cSCC cell invasion, another invasion assay method was used. Collagen gel was prepared in the bottom of the invasion inserts, and the invasion of cSCC cells and fibroblasts through the gel toward the chemoattractant (10% DMEM) was investigated. rhC5a treatment enhanced UT-SCC-7 cell invasion but had no effect on the invasion of fibroblasts compared with the control cultures, confirming the results obtained with the 3D spheroid model (Supplemental Figure S2, I and J).

### C5aR1 Expression on Surface of Tumor Cells in Human cSCC Xenografts

**Figure 3:**
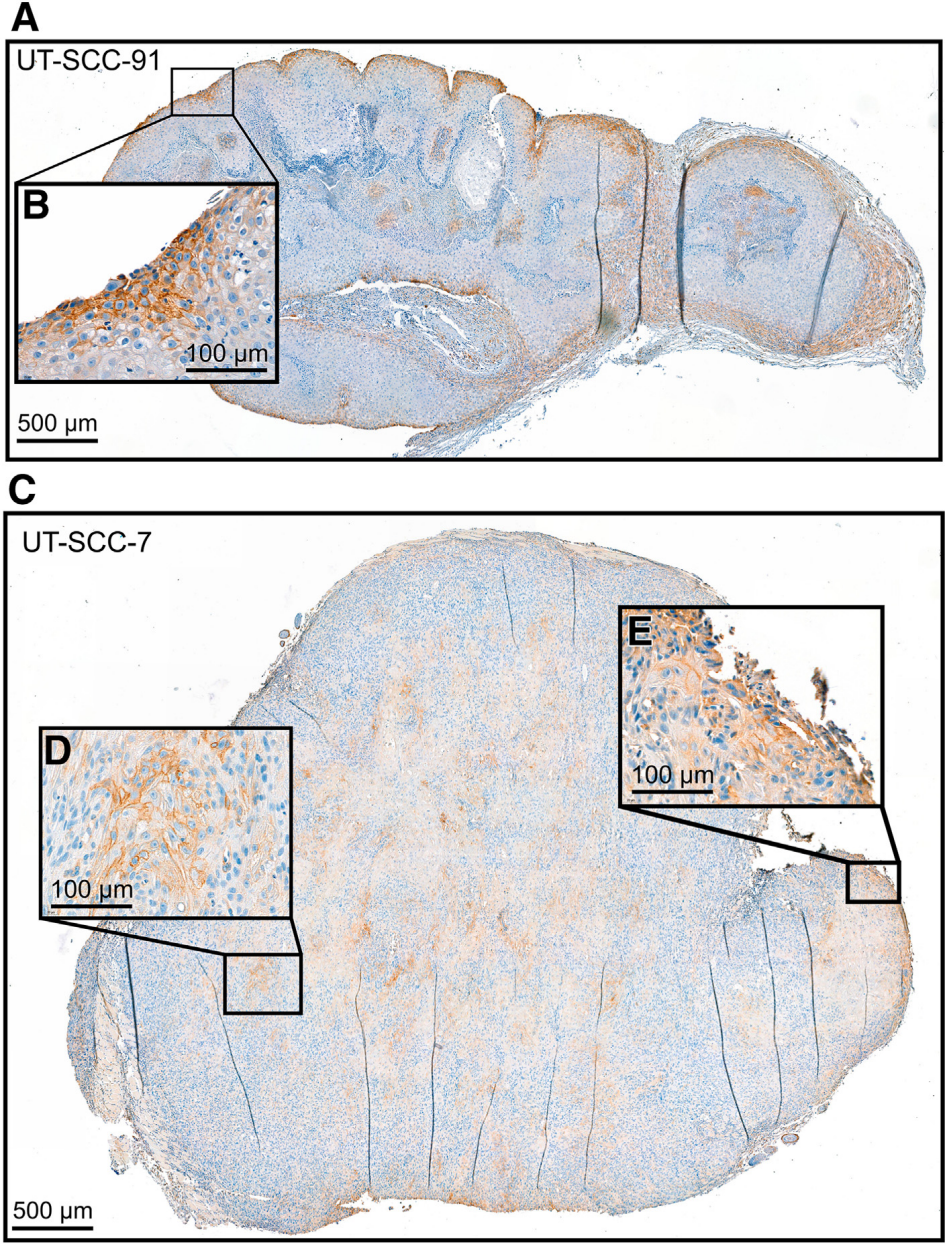
C5aR1 is expressed on tumor cells at the edges of human cutaneous squamous cell carcinoma xenografts. **A** and **C:** Human non-metastatic (UT-SCC-91, 7 × 10^6^) and metastatic (UT-SCC-7, 5 × 10^6^) cutaneous squamous cell carcinoma cell lines were subcutaneously injected into the backs of severe combined immunodeficient mice. The xenograft tumors were analyzed with immunohistochemistry using C5aR1 antibody. **A:** C5aR1 staining in the xenograft tumor established with UT-SCC-91 was predominantly localized at the edges of the xenograft on the tumor cell surface as shown in **B**. **C:** In the xenograft tumor established with UT-SCC-7, C5aR1 staining was observed on the tumor cell surface at the edges as shown in **E**, as well as in the well-differentiated areas within the tumor as represented in **D**. Cell surface staining appeared more heterogeneous within the tumor, with staining primarily located on the cell surface. Scale bars: 100 μm (smaller panels); 500 μm (larger panels).

The expression of C5aR1 *in vivo* was first examined by using a human cSCC xenograft model. IHC staining of xenografts showed C5aR1 on the cSCC cell surface, specifically at the edges of xenograft tumors established with both non-metastatic (UT-SCC-91) (Figure 3, A and B) and metastatic (UT-SCC-7) (Figure 3, C and E) cSCC cell lines. In addition, in the xenograft tumor established with the metastatic UT-SCC-7 cell line, prominent cell surface staining for C5aR1 was also noted in tumor cells in the center of the tumor (Figure 3D).

### Increase in C5aR1-Positive Cells Is Observed in cSCC *in Vivo*

**Figure 4:**
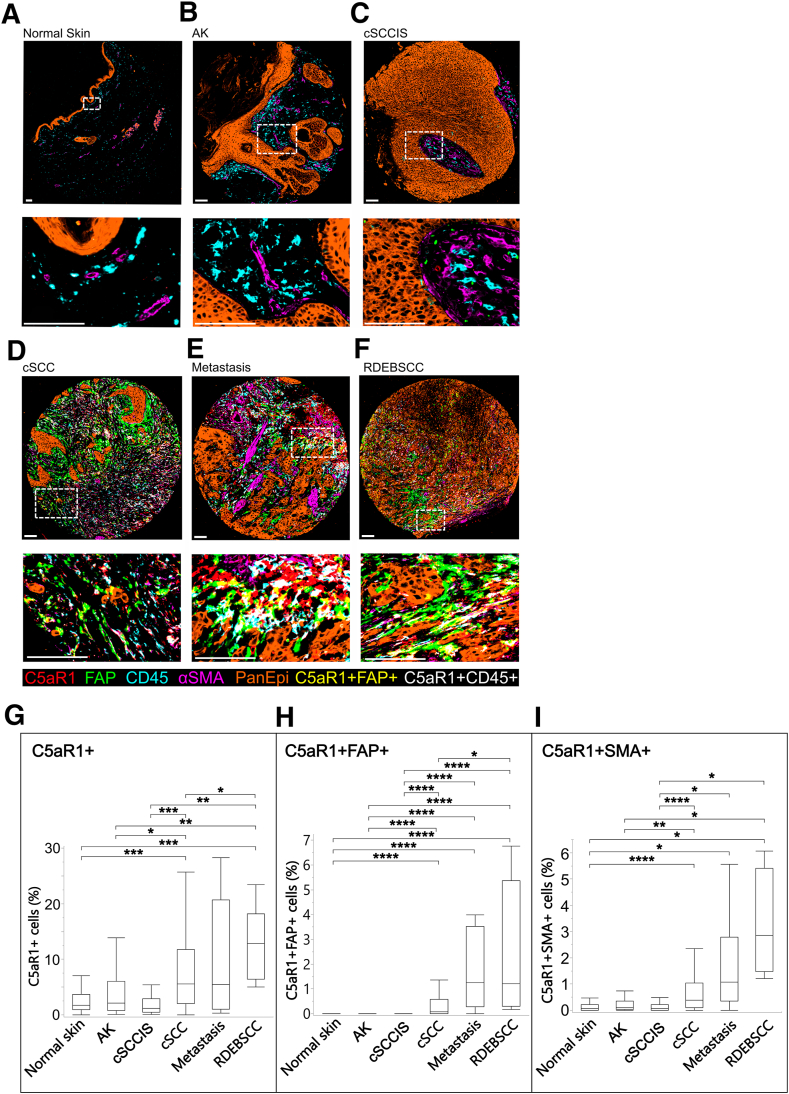
Increased number of C5aR1-positive cells in the cutaneous squamous cell carcinoma (cSCC) tumors. **A–F:** The expression of C5aR1 in normal skin samples (**A**), actinic keratoses (AK) (**B**), cSCC *in situ* (cSCCIS) (**C**), invasive cSCCs (**D**), cSCC metastases (**E**), and recessive dystrophic epidermolysis bullosa–associated cSCC (RDEBSCCs) (**F**) was analyzed by using multiplexed immunofluorescence. Fibroblasts were identified by staining for fibroblast activation protein (FAP) and α-smooth muscle actin (αSMA). PanEpi staining was used to identify epithelial cells and CD45 staining to identify leukocytes. Representative images of the stainings from each group are shown. **G:** The percentage of C5aR1-positive (C5aR1^+^) cells in tissue samples. **H:** The relative number of C5aR1^+^ and FAP-positive (C5aR1^+^FAP^+^) cells in tissue samples. **I:** The relative number of C5aR1^+^ and αSMA-positive (C5aR1^+^SMA^+^) cells in tissue samples. The figures display the maximum, minimum, upper quartile, lower quartile, and median values. *P* values from each pairwise comparison were calculated by using the Steel-Dwass method. *n* = 54 (**A**); *n* = 53 (**B**); *n* = 47 (**C**); *n* = 174 (**D**); *n* = 9 (**E**); *n* = 11 (**F**). ∗*P* < 0.05; ∗∗*P* < 0.01; ∗∗∗*P* < 0.001; ∗∗∗∗*P* < 0.0001. Scale bars = 100 μm (**A–F**). **Boxes areas with white dotted frames** in the top panels are magnified in the bottom panels (**A**–**F**).

The expression of C5aR1 in invasive cSCCs (*n* = 174) (Figure 4D) compared with normal skin (*n* = 62) (Figure 4A), premalignant lesions, AKs (*n* = 53) (Figure 4B), cSCCIS (*n* = 47) (Figure 4C), cSCC metastases (*n* = 9) (Figure 4E), and RDEBSCC (*n* = 11) (Figure 4F) was examined using mIF. The analysis revealed an increased number of C5aR1-positive (C5aR1^+^) cells in invasive cSCCs compared with normal skin and premalignant lesions (Figure 4, A–D). Statistical analysis indicated a significantly higher number of C5aR1^+^ cells in cSCCs compared with normal skin, AK, and cSCCIS (Figure 4G). Multiplex immunofluorescence for FAP and αSMA, two markers for cancer-associated fibroblasts, showed that the relative abundance of C5aR1^+^FAP^+^ cells and C5aR1^+^SMA^+^ cells was higher in cSCCs than in normal skin, AK, and cSCCIS (Figure 4, A–D, H and I). Moreover, the number of C5aR1^+^FAP^+^ and C5aR1^+^SMA^+^ cells in cSCC metastases was similar to those in primary cSCC (Figure 4, H and I). The assessment of samples of RDEBSCC, an aggressive form of cSCC developing in chronic ulcers of patients with RDEB, revealed an elevated number of C5aR1^+^ cells in RDEBSCC compared with cSCC. In addition, the number of C5aR1^+^FAP^+^ cells was higher in RDEBSCC compared with cSCC (Figure 4H).

### C5aR1 Is Overexpressed on the Surface of Metastatic cSCC Cells and Stromal Fibroblasts *in Vivo*

**Figure 5:**
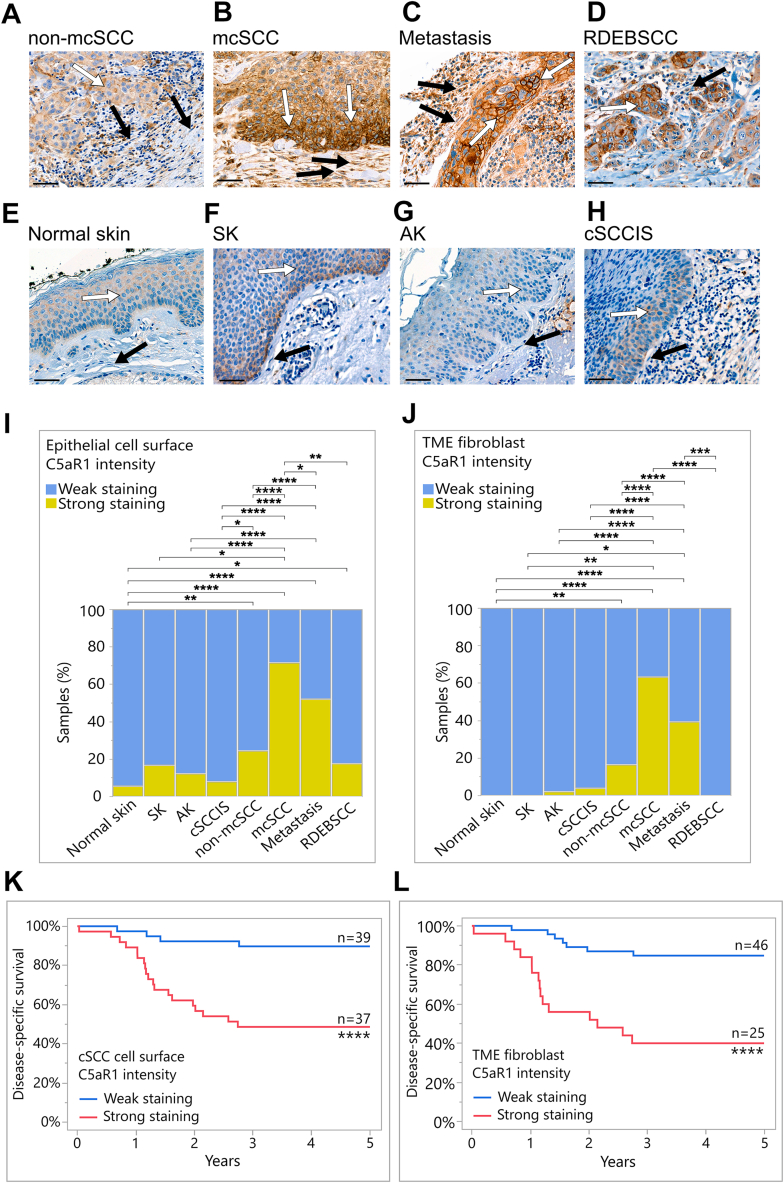
Increased expression of C5aR1 in the cutaneous squamous cell carcinoma (cSCC) *in vivo*. **A–H:** Immunohistochemical staining with a C5aR1 antibody was performed on tissue samples from normal skin (*n* = 54), seborrheic keratosis (SK; *n* = 6), actinic keratosis (AK; *n* = 50), cSCC *in situ* (cSCCIS; *n* = 51), non-metastatic cSCC (non-mcSCC; *n* = 152 samples, 97 individual tumors), metastatic cSCC (mcSCC; *n* = 77 samples, 55 individual tumors), cSCC metastases (*n* = 94 samples, 65 individual tumors), and recessive dystrophic epidermolysis bullosa–associated cSCC (RDEBSCC; *n* = 11). Representative stainings from each group are shown. **B–D:** Increased C5aR1 staining was observed on the surface of cSCC cells, particularly in mcSCC, metastases, and RDEBSCC. **A** and **D–H:** The C5aR1 staining was weaker on the cell surface in normal skin, SK, AK, cSCCIS, and non-mcSCC. In stromal fibroblasts, cytoplasmic C5aR1 staining was strong, especially in mcSCC and metastases shown in panels **B** and **C** compared with normal skin, SK, AK, cSCCIS, RDEBSCC, and non-mSCC shown in panels **A** and **D–H**. **White arrows** indicate tumor and epithelial cells; **black arrows** indicate fibroblasts in the tumor microenvironment (TME). **I** and **J:** C5aR1 immunostaining intensity was scored as weak or strong based on specific staining on the epithelial cell surface and in TME fibroblasts. The C5aR1 staining on the cell surface of cSCC cells and in TME fibroblasts was significantly stronger in mcSCC and metastases compared with normal skin, SK, AK, and cSCCIS. In addition, the staining of C5aR1 was stronger on the cSCC cell surface and in fibroblasts in mcSCC and metastases compared with non-mcSCC. **K** and **L:** The Kaplan-Meier method was used to assess cSCC patient survival. **K:** Disease-specific survival analysis revealed that increased expression of C5aR1 on the tumor cell surface was associated with poor prognosis in patients with cSCC. **L:** Strong C5aR1 staining in TME fibroblasts was also associated with poor prognosis in patients with cSCC. ∗*P* < 0.05, ∗∗*P* < 0.01, ∗∗∗*P* < 0.001, ∗∗∗∗*P* < 0.0001, two-tailed Fisher exact test (**I** and **J**); ∗∗∗∗*P* < 0.0001, log rank test (**K** and **L**). Scale bars: 50 μm (**A–H**).

**Supplemental Figure S3:**
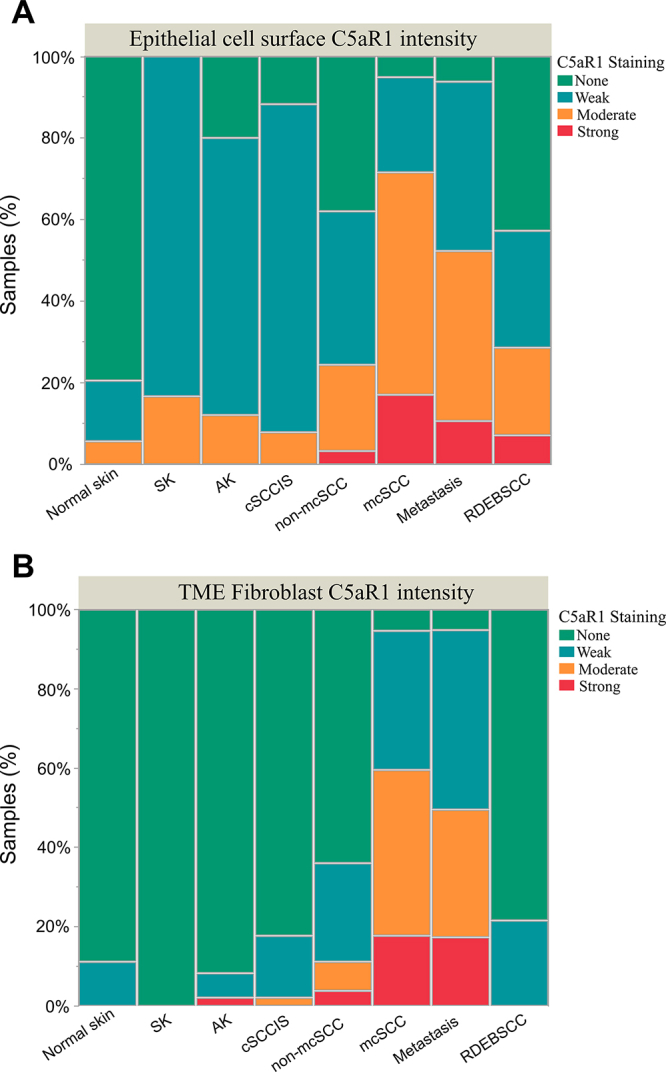
C5aR1 staining on epithelial cell surface and in tumor microenvironment (TME) fibroblasts *in vivo*. **A** and **B:** The original results of semiquantitative analysis of chromogenic immunohistochemical samples showing differences in C5aR1 staining on the cutaneous squamous cell carcinoma (cSCC) cell surface and in TME fibroblasts in normal skin (*n* = 54), seborrheic keratosis (SK; *n* = 6), actinic keratosis (AK; *n* = 50), cSCC *in situ* (cSCCIS; *n* = 51), non-metastatic cSCC (non-mcSCC; *n* = 152 samples, 97 individual tumors), metastatic cSCC (mcSCC; *n* = 77 samples, 55 individual tumors), cSCC metastases (*n* = 94 samples, 65 individual tumors), and recessive dystrophic epidermolysis bullosa–associated cSCC (RDEBSCC; *n* = 11).

To further explore the expression and localization of C5aR1, tissue microarrays containing tissue samples of normal skin, SK, AK, cSCCIS, primary non-mcSCC and mcSCC, metastasis, and RDEBSCC were stained with a C5aR1 antibody using chromogenic IHC. C5aR1 staining was specifically noted on the surface of epithelial cells and with increased staining in cSCC compared with normal skin, SK, AK, or cSCCIS (Figure 5). Moreover, increased C5aR1 staining on the tumor cell surface was evident in mcSCC (Figure 5B) and cSCC metastases (Figure 5C) compared with non-mcSCC (Figure 5A). In addition, more abundant C5aR1 staining was observed on the tumor cell surface in RDEBSCC (Figure 5D) compared with normal skin (Figure 5E). Interestingly, C5aR1 expression was detected in stromal fibroblasts located near the tumor (Figure 5, B and C). For semiquantitative analysis, only fibroblasts in the reticular dermis were included. C5aR1 staining was significantly stronger in TME fibroblasts in mcSCCs compared with normal skin, AK, cSCCIS, and non-mcSCC samples (Figure 5, A, B, E, G, H, and J, and Supplemental Figure S3). In normal skin and SK samples, all analyzed fibroblasts were negative (Figure 5, E, F, and J). In addition, C5aR1 staining on the tumor cell surface and in TME fibroblasts was increased in mcSCC and cSCC metastases compared with non-mcSCC (Figure 5, A, B, C, I, and J, and Supplemental Figure S3).

**Supplemental Figure S4:**
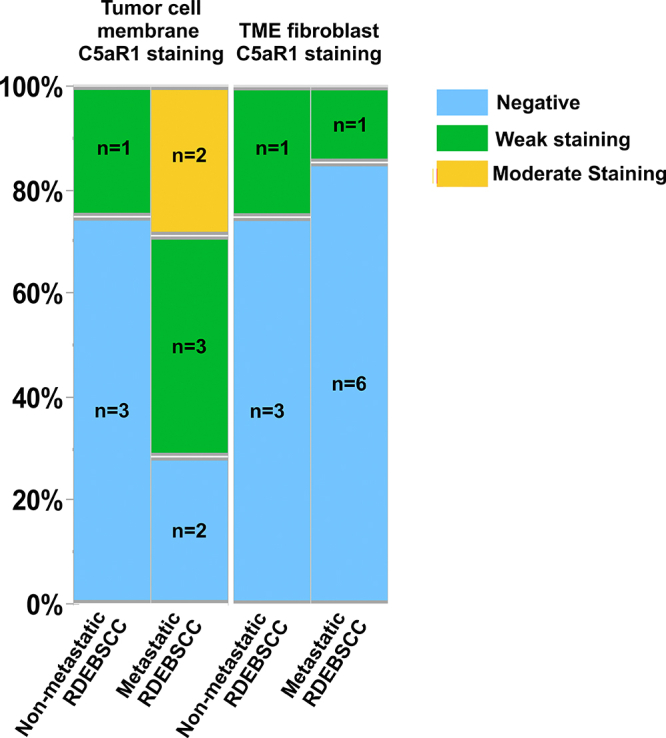
C5aR1 staining on tumor cell surface and in tumor microenvironment (TME) fibroblasts in non-metastatic and metastatic recessive dystrophic epidermolysis bullosa–associated cutaneous squamous cell carcinoma (RDEBSCC) tumors. The original results of semiquantitative analysis of chromogenic immunohistochemical staining show differences in C5aR1 staining on the cutaneous squamous cell carcinoma cell surface and in TME fibroblasts in non-metastatic (*n* = 4) and metastatic (*n* = 7) RDEBSCC tumors.

In the semiquantitative analysis of RDEBSCC tumors (Supplemental Figure S4), a total of seven metastatic and four non-metastatic tumors were evaluated. Among the metastatic RDEBSCC samples, two had moderately positive C5aR1 expression on the cell surface of tumor cells, three showed weak staining, and two were negative. In comparison, only one of the four non-metastatic tumors exhibited weak positivity, with the remaining three showing no detectable staining. In the fibroblasts of RDEBSCC tumors, weak C5aR1 staining was observed in one of the four non-metastatic tumors, whereas the other three were negative. Among the metastatic cases, weak staining was noted in seven tumors, with the remaining six showing no staining.

### Increased Expression of C5aR1 in cSCC *in Vivo* Is Associated with Metastasis and Poor Prognosis

**Supplemental Figure S5:**
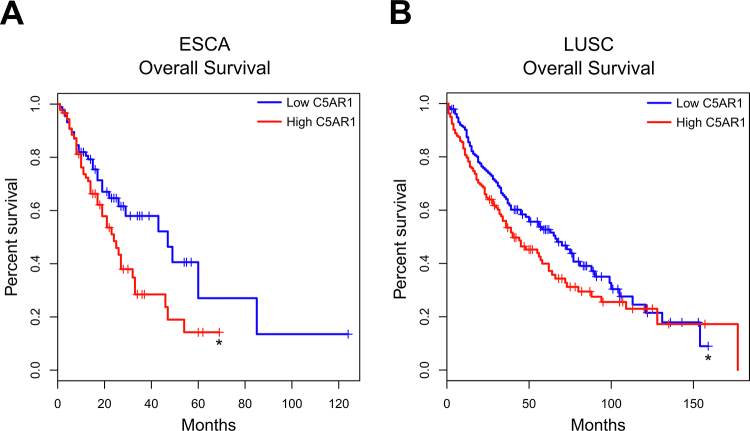
Correlation of the expression of C5AR1 mRNA with prognosis of the patients with esophageal carcinoma (ESCA) and lung squamous cell carcinoma (LUSC). **A** and **B:** Evaluation of the impact of C5AR1 on the overall survival of ESCA and LUSC patients was performed by using the GEPIA tool with data available from The Cancer Genome Atlas. Median level was used as the basis of defining low and high C5AR1. ∗*P* < 0.05.

The semiquantitative analysis of C5aR1 IHC stainings was extended to include survival analysis. The disease-specific 5-year survival analysis revealed that the elevated expression of C5aR1 on the cSCC cell surface (Figure 5K) and in fibroblasts within the TME (Figure 5L), was associated with poor prognosis in patients with cSCC. Moreover, this poor prognosis was already evident at 3 years (Figure 5, K and L). Furthermore, the impact of C5aR1 on the overall survival of patients with other SCCs was assessed by using The Cancer Genome Atlas database. Elevated expression of C5AR1 correlated with a poor prognosis, resulting in shorter overall survival in esophageal carcinoma and lung SCC (Supplemental Figure S5).

## Discussion

The complement cascade is part of the human innate immunity system and has conventionally been viewed as a tumor-suppressing cytolytic mechanism. However, numerous studies have shown that complement components and their receptors also contribute to tumor progression and metastasis by inducing inflammation or causing immunosuppression.^8^, ^9^, ^10^ Activation of the complement cascade leads to the cleavage of the complement molecule C5 into C5a and C5b. C5a acts as an anaphylatoxin, whereas C5b is involved in the complement lytic pathway. C5a binds to two receptors [C5aR1 and C5aR2 (C5L2)] on the surfaces of phagocytes and other cell types.^19^^,^^20^ The role of C5aR1 in the inflammatory response is well established, whereas the function of C5aR2 is less well understood.^20^ In addition, the role of the C5a-C5aR1 axis in the progression of cSCC is not known.

The aim of the current study was to investigate the role of C5aR1 in the progression and metastasis of cSCC. In previous studies, the expression of C5aR1 at the mRNA level was not detected in cSCC cells cultured in a monolayer.^12^ Here, low expression of C5aR1 at the protein level was observed in an mcSCC cell line (UT-SCC-7) cultured in 3D spheroids. However, when cSCC cells were cocultured with normal dermal fibroblasts in spheroids, the protein-level expression of C5aR1 in cSCC cells increased. Furthermore, treatment of Ha-*ras*-transformed metastatic human keratinocytes, RT3 cells, cocultured in spheroids with human skin fibroblasts with the C5aR1 ligand rhC5a significantly enhanced the invasion of RT3, UT-SCC-115, and UT-SCC-7 cells through collagen I. These results suggest that the interaction between fibroblasts and cancer cells is necessary to induce the expression of C5aR1 by cSCC cells and that C5aR1 promotes the invasion of cSCC cells. This observation is further supported by the C5aR1 expression noted *in vivo* at the surface of cSCC cells at the invading margin in xenograft tumors established with non-mcSCC and mcSCC cell lines. Furthermore, these findings are consistent with previous research on the role of C5aR1 in promoting the invasion of gastric cancer cells.^46^

The expression of C5aR1 was examined in a large panel of tissue samples representing the progression from AKs to invasive and mcSCC, as well as metastases using mIF and chromogenic IHC. C5aR1 expression was observed on the surface of cSCC tumor cells *in vivo*. C5aR1 expression was significantly higher in cSCC compared with that in AKs, cSCCIS, normal skin, or benign papillomas (SKs). Moreover, C5aR1 expression was increased in mcSCC compared with non-mcSCC, and the expression was also significantly higher in metastases than in non-mcSCC. Notably, mIF visualizes the relative cell area positive for C5aR1, and chromogenic IHC indicates the localization and intensity of the staining. These results suggest that C5aR1 may serve as a marker for metastatic primary cSCCs or cSCC metastases. Similar findings have been observed in lung cancer, and meta-analyses have shown a higher level of C5aR1 associated with the occurrence of lymph node metastases.^33^^,^^47^, ^48^, ^49^ Interestingly, in RDEBSCCs, the expression of C5aR1 on cSCC tumor cells was comparable to that in non-mcSCC, whereas lesser expression was noted in TME fibroblasts. This finding suggests that although RDEBSCC is an aggressive form of cSCC, there are differences in the expression of C5aR1^+^ fibroblasts compared with sporadic cSCCs. These findings are in accordance with our recent observations showing differences in the cancer-associated fibroblast (CAF) population between cSCC and RDEBSCC.^45^

The role of CAFs in cancer progression has recently received attention, and the role of CAFs in cancer and cSCC have been emphasized.^50^^,^^51^ Recent observations indicate that FAP- and αSMA-positive CAFs are increased in invasive cSCCs.^45^ The findings of this study show the expression of C5aR1 by TME fibroblasts in cSCC, with an increased co-localization of FAP- and αSMA-positive fibroblasts compared with normal skin, AKs, and cSCCIS. These results align with previous studies showing that the activation of C3a-C3aR signaling in a mouse breast cancer model results in enhanced lung metastasis formation by modulating CAFs.^52^

C5a increased the invasiveness of cSCC cells, and an up-regulation in the expression of C5aR1 was also observed on the tumor cell surface as well as in CAFs. These results are in accordance with observations in other cancers, such as lung cancer,^47^, ^48^, ^49^ and suggest that the C5a-C5aR1 pathway likely contributes to promoting metastasis in cSCC as well. In addition, the current results indicated that high expression of C5aR1 correlated with a poor prognosis. These findings highlight the potential value of C5aR1 expression as a prognostic marker for cSCC, and C5aR1 staining could serve as a predictive biomarker for the prognosis of patients with cSCC.

Knocking down the anaphylatoxin-related pathway can result in antitumoral effects in many cancers.^34^ The potential mechanisms that may underlie the correlation between the metastatic potential of cSCC and the overexpression of C5aR1 could be linked to the established functions of anaphylatoxins. Anaphylatoxins are vasodilators and increase the vascular permeability.^18^ This function of anaphylatoxins could enhance the metastatic potential of cSCC tumors by increasing the permeability of capillaries in the TME. Anaphylatoxins can also serve as promoters of chronic inflammation in the TME, thereby promoting tumor progression.^8^, ^9^, ^10^

The C5a-C5aR1 signaling pathway plays an important role in the inflammatory cell signaling within the TME, and this pathway has been recognized as a potential therapeutic target in the context of checkpoint inhibition.^47^^,^^48^ Inhibiting C5a-C5aR signaling improves the efficacy of programmed cell death protein 1 blockade, resulting in a significant reduction in tumor growth and metastasis, along with prolonged survival in patients with lung cancer.^47^ This finding is particularly intriguing in the context of treating advanced cSCC.

Currently, there are three immune-oncologic antibody treatments available for locally advanced and mcSCCs targeting the programmed cell death protein 1/programmed death ligand 1 pathway. They are cemiplimab, which is approved by the US Food and Drug Administration and the European Medicines Agency, and pembrolizumab and cosibelimab, which are approved by the US Food and Drug Administration.^53^, ^54^, ^55^, ^56^ The findings of the current study suggest that the C5a-C5aR1 axis could potentially serve as a therapeutic target in cSCC in combination with programmed cell death protein 1 antibody treatment. Currently, three antibodies against C5a and one small-molecule inhibitor against C5aR1 have been approved for the treatment of inflammatory diseases.^57^ It is conceivable that these therapies could also represent viable options for treating solid cancers, including advanced cSCC.

In conclusion, the current results show elevated C5aR1 expression in cSCC tumors, particularly at the invasive tumor edges and in stromal fibroblasts, compared with normal skin, benign papillomas (SKs), AKs, or cSCCIS. C5a promoted cSCC cell invasion, and the expression of C5aR1 was linked to metastatic risk and poor prognosis in patients with cSCC. These findings suggest that C5aR1 could serve as a potential metastatic risk marker, a novel prognostic biomarker, and promising therapeutic target for cSCC.

## Declaration of Generative AI and AI-Assisted Technologies in the Writing Process

During the preparation of this work, the authors used ChatGPT-4 (OpenAI, San Francisco, CA) to revise the language of some paragraphs during the final editing of the manuscript. After using this tool/service, the authors reviewed and edited the content as needed, and take full responsibility for the content of the publication.

## Disclosure Statement

None declared.

## References

[1] Nehal K.S., Bichakjian C.K. (2018). Update on keratinocyte carcinomas. N Engl J Med.

[2] Venables Z.C., Autier P., Nijsten T., Wong K.F., Langan S.M., Rous B., Broggio J., Harwood C., Henson K., Proby C.M., Rashbass J., Leigh I.M. (2019). Nationwide incidence of metastatic cutaneous squamous cell carcinoma in England. JAMA Dermatol.

[3] Venables Z.C., Nijsten T., Wong K.F., Autier P., Broggio J., Deas A., Harwood C.A., Hollestein L.M., Langan S.M., Morgan E., Proby C.M., Rashbass J., Leigh I.M. (2019). Epidemiology of basal and cutaneous squamous cell carcinoma in the U.K. 2013-15: a cohort study. Br J Dermatol.

[4] Knuutila J.S., Kaijala O., Lehto S., Vahlberg T., Nissinen L., Kähäri V.-M., Riihilä P. (2024). Clinical risk factors for cutaneous squamous cell carcinoma in patients with actinic keratosis or cutaneous squamous cell carcinoma in situ: a retrospective double-cohort study. Acta Derm Venereol.

[5] Knuutila J., Riihilä P., Kurki S., Nissinen L., Kähäri V.-M. (2020). Risk factors and prognosis for metastatic cutaneous squamous cell carcinoma: A cohort study. Acta Derm Venereol.

[6] Nagarajan P., Asgari M.M., Green A.C., Guhan S.M., Arron S.T., Proby C.M., Rollison D.E., Harwood C.A., Toland A.E. (2019). Keratinocyte carcinomas: current concepts and future research priorities. Clin Cancer Res.

[7] Stratigos A.J., Garbe C., Dessinioti C., Lebbe C., Akkooi A., Bataille V., Bastholt L., Dreno B., Dummer R., Fargnoli M.C., Forsea A.M., Harwood C.A., Hauschild A., Hoeller C., Kandolf-Sekulovic L., Kaufmann R., Kelleners-Smeets N.W.J., Lallas A., Leiter U., Malvehy J., Del Marmol V., Moreno-Ramirez D., Pellacani G., Peris K., Saiag P., Tagliaferri L., Trakatelli M., Ioannides D., Vieira R., Zalaudek I., Arenberger P., Eggermont A.M.M., Röcken M., Grob J.-J., Lorigan P. (2023). European consensus-based interdisciplinary guideline for invasive cutaneous squamous cell carcinoma. Part 1: diagnostics and prevention—update 2023. Eur J Cancer.

[8] Pio R., Corrales L., Lambris J.D. (2014). The role of complement in tumor growth. Adv Exp Med Biol.

[9] Riihilä P., Nissinen L., Knuutila J., Rahmati Nezhad P., Viiklepp K., Kähäri V.-M. (2019). Complement system in cutaneous squamous cell carcinoma. Int J Mol Sci.

[10] Meri S., Magrini E., Mantovani A., Garlanda C. (2023). The yin yang of complement and cancer. Cancer Immunol Res.

[11] Riihilä P.M., Nissinen L.M., Ala-Aho R., Kallajoki M., Grénman R., Meri S., Peltonen S., Peltonen J., Kähäri V.-M. (2014). Complement factor H: a biomarker for progression of cutaneous squamous cell carcinoma. J Invest Dermatol.

[12] Riihilä P., Nissinen L., Farshchian M., Kivisaari A., Ala-Aho R., Kallajoki M., Grénman R., Meri S., Peltonen S., Peltonen J., Kähäri V.-M. (2015). Complement factor I promotes progression of cutaneous squamous cell carcinoma. J Invest Dermatol.

[13] Riihilä P., Nissinen L., Farshchian M., Kallajoki M., Kivisaari A., Meri S., Grénman R., Peltonen S., Peltonen J., Pihlajaniemi T., Heljasvaara R., Kähäri V.-M. (2017). Complement component C3 and complement factor B promote growth of cutaneous squamous cell carcinoma. Am J Pathol.

[14] Riihilä P., Viiklepp K., Nissinen L., Farshchian M., Kallajoki M., Kivisaari A., Meri S., Peltonen S., Peltonen J., Kähäri V.-M. (2020). Tumour-cell-derived complement components C1r and C1s promote growth of cutaneous squamous cell carcinoma. Br J Dermatol.

[15] Rahmati Nezhad P., Riihilä P., Piipponen M., Kallajoki M., Meri S., Nissinen L., Kähäri V.-M. (2021). Complement factor I upregulates expression of matrix metalloproteinase-13 and -2 and promotes invasion of cutaneous squamous carcinoma cells. Exp Dermatol.

[16] Viiklepp K., Nissinen L., Ojalill M., Riihilä P., Kallajoki M., Meri S., Heino J., Kähäri V.-M. (2022). C1r upregulates production of matrix metalloproteinase-13 and promotes invasion of cutaneous squamous cell carcinoma. J Invest Dermatol.

[17] Rahmati Nezhad P., Riihilä P., Knuutila J.S., Viiklepp K., Peltonen S., Kallajoki M., Meri S., Nissinen L., Kähäri V.-M. (2022). Complement factor D is a novel biomarker and putative therapeutic target in cutaneous squamous cell carcinoma. Cancers (Basel).

[18] Peng Q., Li K., Sacks S.H., Zhou W. (2009). The role of anaphylatoxins C3a and C5a in regulating innate and adaptive immune responses. Inflamm Allergy Drug Targets.

[19] Lee H., Whitfeld P.L., Mackay C.R. (2008). Receptors for complement C5a. The importance of C5aR and the enigmatic role of C5L2. Immunol Cel Biol.

[20] Ward P.A. (2009). Functions of C5a receptors. J Mol Med (Berl).

[21] Huber-Lang M.S., Younkin E.M., Sarma J.V., McGuire S.R., Lu K.T., Guo R.F., Padgaonkar V.A., Curnutte J.T., Erickson R., Ward P.A. (2002). Complement-induced impairment of innate immunity during sepsis. J Immunol.

[22] Sarma V.J., Huber-Lang M., Ward P.A. (2006). Complement in lung disease. Autoimmunity.

[23] Hopkins P., Belmont H.M., Buyon J., Philips M., Weissmann G., Abramson S.B. (1988). Increased levels of plasma anaphylatoxins in systemic lupus erythematosus predict flares of the disease and may elicit vascular injury in lupus cerebritis. Arthritis Rheum.

[24] Gu J., Ding J.-Y., Lu C.-L., Lin Z.-W., Chu Y.-W., Zhao G.-Y., Guo J., Ge D. (2013). Overexpression of CD88 predicts poor prognosis in non-small-cell lung cancer. Lung Cancer.

[25] Wada Y., Maeda Y., Kubo T., Kikuchi K., Eto M., Imamura T. (2016). C5a receptor expression is associated with poor prognosis in urothelial cell carcinoma patients treated with radical cystectomy or nephroureterectomy. Oncol Lett.

[26] Maeda Y., Kawano Y., Wada Y., Yatsuda J., Motoshima T., Murakami Y., Kikuchi K., Inamura T., Eto M. (2015). C5aR is frequently expressed in metastatic renal cell carcinoma and plays a crucial role in cell invasion via the ERK and PI3 kinase pathways. Oncol Rep.

[27] Xi W., Liu L., Wang J., Xia Y., Bai Q., Xiong Y., Yang Q., Long Q., Xu J., Guo J. (2016). Enrichment of C5a-C5aR axis predicts poor postoperative prognosis of patients with clear cell renal cell carcinoma. Oncotarget.

[28] Kaida T., Nitta H., Kitano Y., Yamamura K., Arima K., Izumi D., Higashi T., Kurashige J., Imai K., Hayashi H., Iwatsuki M., Ishimoto T., Hashimoto D., Yamashita Y., Chikamoto A., Imanura T., Ishiko T., Beppu T., Baba H. (2016). C5a receptor (CD88) promotes motility and invasiveness of gastric cancer by activating RhoA. Oncotarget.

[29] Nitta H., Shimose T., Emi Y., Imamura T., Ohnishi K., Kusumoto T., Yamamoto M., Fukuzawa K., Takahashi I., Higashi H., Tsuji A., Akagi Y., Oki E., Maehara Y., Baba H., Kyushu Study Group of Clinical Care (KSCC) ancillary study (2016). Expression of the anaphylatoxin C5a receptor in gastric cancer: implications for vascular invasion and patient outcomes. Med Oncol.

[30] Hu W.-H., Hu Z., Shen X., Dong L.-Y., Zhou W.-Z., Yu X.-X. (2016). C5a receptor enhances hepatocellular carcinoma cell invasiveness via activating ERK1/2-mediated epithelial-mesenchymal transition. Exp Mol Pathol.

[31] Imamura R., Kitagawa S., Kubo T., Irie A., Kariu T., Yoneda M., Kamba T., Imamura T. (2021). Prostate cancer C5a receptor expression and augmentation of cancer cell proliferation, invasion, and PD-L1 expression by C5a. Prostate.

[32] Imamura T., Yamamoto-Ibusuki M., Sueta A., Kubo T., Irie A., Kikuchi K., Kariu T., Iwase H. (2016). Influence of the C5a-C5a receptor system on breast cancer progression and patient prognosis. Breast Cancer.

[33] Wang Z., Yu W., Qiang Y., Ma F., Ding P., Wang Y. (2021). Clinicopathological features and prognostic significance of C5aR in human solid tumors: a meta-analysis. BMC Cancer.

[34] Ajona D., Ortiz-Espinosa S., Pio R. (2019). Complement anaphylatoxins C3a and C5a: emerging roles in cancer progression and treatment. Semin Cel Dev Biol.

[35] Roumenina L.T., Daugan M.V., Petitprez F., Sautès-Fridman C., Fridman W.H. (2019). Context-dependent roles of complement in cancer. Nat Rev Cancer.

[36] Farshchian M., Nissinen L., Grénman R., Kähäri V.-M. (2017). Dasatinib promotes apoptosis of cutaneous squamous carcinoma cells by regulating activation of ERK1/2. Exp Dermatol.

[37] Nissinen L., Riihilä P., Viiklepp K., Rajagopal V., Storek M., Kähäri V.-M. (2024). C1s targeting antibodies inhibit the growth of cutaneous squamous carcinoma cells. Sci Rep.

[38] Boukamp P., Stanbridge E.J., Foo D.Y., Cerutti P.A., Fusenig N.E. (1990). c-Ha-ras oncogene expression in immortalized human keratinocytes (HaCaT) alters growth potential in vivo but lacks correlation with malignancy. Cancer Res.

[39] Siljamäki E., Rappu P., Riihilä P., Nissinen L., Kähäri V.-M., Heino J. (2020). H-Ras activation and fibroblast-induced TGF-[beta] signaling promote laminin-332 accumulation and invasion in cutaneous squamous cell carcinoma. Matrix Biol.

[40] Siljamäki E., Riihilä P., Suwal U., Nissinen L., Rappu P., Kallajoki M., Kähäri V.-M., Heino J. (2023). Inhibition of TGF-[beta] signaling, invasion, and growth of cutaneous squamous cell carcinoma by PLX8394. Oncogene.

[41] Peirsman A., Blondeel E., Ahmed T., Anckaert J., Audenaert D., Boterberg T. (2021). MISpheroID: a knowledgebase and transparency tool for minimum information in spheroid identity. Nat Methods.

[42] Schindelin J., Arganda-Carreras I., Frise E., Kaynig V., Longair M., Pietzsch T., Preibisch S., Rueden C., Saalfeld S., Schmid B., Tinevez J.-Y., White D.J., Hartenstein V., Eliceiri K., Tomancak P., Cardona A. (2012). Fiji: an open-source platform for biological-image analysis. Nat Methods.

[43] Uhlen M., Zhang C., Lee S., Sjöstedt E., Fagerberg L., Bidkhori G., Benfeitas R., Arif M., Liu Z., Edfors F., Sanli K., von Feilitzen K., Oksvold P., Lundberg E., Hober S., Nilsson P., Mattsson J., Schwenk J.M., Brunnström H., Glimelius B., Sjöblom T., Edqvist P.-H., Djureinovic D., Micke P., Lindskog C., Mardinoglu A., Pontén F. (2017). A Pathology Atlas Of The Human Cancer Transcriptome. Science.

[44] Weinstein J.N., Collisson E.A., Mills G.B., Mills Shaw K.R., Ozenberger B.A., Ellrott K., Shmulevich I., Sander C., Stuart J.M., Cancer Genome Atlas Research Network (2013). : The Cancer Genome Atlas Pan-Cancer analysis project. Nat Genet.

[45] Knuutila J.S., Riihilä P., Nissinen L., Heiskanen L., Kallionpää R.E., Pellinen T., Kähäri V.-M. (2024). Cancer-associated fibroblast activation predicts progression, metastasis, and prognosis of cutaneous squamous cell carcinoma. Int J Cancer.

[46] Nitta H., Wada Y., Kawano Y., Murakami Y., Irie A., Taniguchi K., Kikuchi K., Yamada G., Suzuki K., Honda J., Wilson-Morifuji M., Araki N., Eto M., Baba H., Imamura T. (2013). Enhancement of human cancer cell motility and invasiveness by anaphylatoxin C5a via aberrantly expressed C5a receptor (CD88). Clin Cancer Res.

[47] Ajona D., Ortiz-Espinosa S., Moreno H., Lozano T., Pajares M.J., Agorreta J., Bértolo C., Lasarte J.J., Vicent S., Hoehlig K., Vater A., Lecanda F., Montuenga L.M., Pio R. (2017). A combined PD-1/C5a blockade synergistically protects against lung cancer growth and metastasis. Cancer Discov.

[48] Ajona D., Zandueta C., Corrales L., Moreno H., Pajares M.J., Ortiz-Espinosa S., Martínez-Terroba E., Perurena N., de Miguel F.J., Jantus-Lewintre E., Camps C., Vicent S., Agorreta J., Montuenga L.M., Pio R., Lecanda F. (2018). Blockade of the complement C5a/C5aR1 axis impairs lung cancer bone metastasis by CXCL16-mediated effects. Am J Respir Crit Care Med.

[49] Vadrevu S.K., Chintala N.K., Sharma S.K., Sharma P., Cleveland C., Riediger L., Manne S., Fairlie D.P., Gorczyca W., Almanza O., Karbowniczek M., Markiewski M.M. (2014). Complement c5a receptor facilitates cancer metastasis by altering T-cell responses in the metastatic niche. Cancer Res.

[50] Ng Y.-Z., Pourreyron C., Salas-Alanis J.C., Dayal J.H.S., Cepeda-Valdes R., Yan W., Wright S., Chen M., Fine J.-D., Hogg F.J., McGrath J.A., Murrell D.F., Leigh I.M., Lane E.B., South A.P. (2012). Fibroblast-derived dermal matrix drives development of aggressive cutaneous squamous cell carcinoma in patients with recessive dystrophic epidermolysis bullosa. Cancer Res.

[51] Sahai E., Astsaturov I., Cukierman E., DeNardo D.G., Egeblad M., Evans R.M., Fearon D., Greten F.R., Hingorani S.R., Hunter T., Hynes R.O., Jain R.K., Janowitz T., Jorgensen C., Kimmelman A.C., Kolonin M.G., Maki R.G., Powers R.S., Puré E., Ramirez D.C., Scherz-Shouval R., Sherman M.H., Stewart S., Tlsty T.D., Tuveson D.A., Watt F.M., Weaver V., Weeraratna A.T., Werb Z. (2020). A framework for advancing our understanding of cancer-associated fibroblasts. Nat Rev Cancer.

[52] Shu C., Zha H., Long H., Wang X., Yang F., Gao J., Hu C., Zhou L., Guo B., Zhu B. (2020). C3a-C3aR signaling promotes breast cancer lung metastasis via modulating carcinoma-associated fibroblasts. J Exp Clin Cancer Res.

[53] Migden M.R., Rischin D., Schmults C.D., Guminski A., Hauschild A., Lewis K.D. (2018). PD-1 blockade with cemiplimab in advanced cutaneous squamous cell carcinoma. N Engl J Med.

[54] Peris K., Piccerillo A., Del Regno L., Di Stefani A. (2022). Treatment approaches of advanced cutaneous squamous cell carcinoma. J Eur Acad Dermatol Venereol.

[55] Stratigos A.J., Garbe C., Dessinioti C., Lebbe C., van Akkooi A., Bataille V., Bastholt L., Dreno B., Dummer R., Fargnoli M.C., Forsea A.M., Harwood C.A., Hauschild A., Hoeller C., Kandolf-Sekulovic L., Kaufmann R., Kelleners-Smeets N.W.J., Lallas A., Leiter U., Malvehy J., Del Marmol V., Moreno-Ramirez D., Pellacani G., Peris K., Saiag P., Tagliaferri L., Trakatelli M., Ioannides D., Vieira R., Zalaudek I., Arenberger P., Eggermont A.M.M., Röcken M., Grob J.-J., Lorigan P. (2023). European consensus-based interdisciplinary guideline for invasive cutaneous squamous cell carcinoma. Part 2: treatment—update 2023. Eur J Cancer.

[56] Clingan P., Ladwa R., Brungs D., Harris D.L., McGrath M., Arnold S., Coward J., Fourie S., Kurochkin A., Malan D.R., Mant A., Sharma V., Shue H., Tazbirkova A., Berciano-Guerrero M.A., Charoentum C., Dalle S., Dechaphunkul A., Dudnichenko O., Koralewski P., Lugowska I., Montaudié H., Muñoz-Couselo E., Sriuranpong V., Oliviero J., Desai J. (2023). Efficacy and safety of cosibelimab, an anti-PD-L1 antibody, in metastatic cutaneous squamous cell carcinoma. J Immunother Cancer.

[57] West E.E., Woodruff T., Fremeaux-Bacchi V., Kemper C. (2024). Complement in human disease: approved and up-and-coming therapeutics. Lancet.

