# Supplementary material for: C5aR1 Promotes Invasion, Metastasis, and Poor Prognosis in Cutaneous Squamous Cell Carcinoma
Source: Am J Pathol. 2025 Mar 6;195(6):1158–71. doi: 10.1016/j.ajpath.2025.02.004 (PMC12163391; doi:10.1016/j.ajpath.2025.02.004)
Supplement: Supplementary Table S1 [file mmc1.docx]

**Supplemental Table S1. Baseline characteristics of cSCC patients and tumors.**

| **cSCC patient characteristics** | **Total**  n=143 patients | **non-mcSCC**  n=90 patients | **mcSCC**  n=53 patients |
| --- | --- | --- | --- |
| **Age (y), mean (min, max)** | 77.3 (27, 102) | 79.3 (47,102) | 74.4 (27, 93) |
| **Sex**  **Male, n (%)**  **Female, n (%)** | 90 (59.6)  61 (40.4) | 42 (56.0)  33 (44.0) | 34 (65.4)  18 (34.6) |

| **cSCC tumor characteristics** | **Total**  n=152 tumors | **non-mcSCC**  n= 97 tumors | **mcSCC**  n= 55 tumors |
| --- | --- | --- | --- |
| **Location**  **Head and neck, n (%)**  **Upper limb, n (%)**  **Lower limb, n (%)**  **Torso, n (%)** | 127 (83.6)  13 (8.6)  9 (5.9)  3 (2.0) | 84 (86.6)  7 (7.2)  4 (4.1)  2 (2.1) | 43 (78.2)  6 (10.9)  5 (9.1)  1 (1.8) |
| **Diameter (mm), Md (Q1, Q3)** | 15 (9, 30) | 11 (9, 20) | 28 (15, 40) |
| **Differentiation**  **Good, n (%)**  **Moderate, n (%)**  **Poor, n (%)**  **Missing, n (%)** | 55 (36.2)  65 (42.8)  30 (19.7)  2 (1.3) | 44 (45.4)  36 (37.1)  15 (15.5)  2 (2.3) | 11 (20.0)  29 (52.7)  15 (27.3)  0 (0) |
| **Necrosis among primary tumors**  **No, n (%)**  **Yes, n (%)**  **Missing, n (%)** | 134 (88.2)  15 (9.8)  3 (3.0) | 90 (92.8)  6 (6.2)  1 (1.0) | 44 (80.0)  9 (16.4)  2 (3.6) |
| **Clark’s level**  **2-4, n (%)**  **5, n (%)**  **Missing, n (%)** | 68 (44.8)  80 (52.6)  4 (2.6) | 66 (68.0)  29 (29.9)  2 (2.1) | 2 (3.6)  51 (92.8)  2 (3.6) |
| **Invasion beyond fat**  **No, n (%)**  **Yes, n (%)**  **Missing, n (%)** | 102 (67.1)  49 (32.2)  1 (0.7) | 82 (84.5)  15 (15.5)  0 (0.0) | 20 (36.4)  34 (61.8)  1 (1.8) |
| **AJCC-8**  **T1, n (%)**  **T2, n (%)**  **T3, n (%)**  **T4a-T4b, n (%)**  **TX, n (%)** | 58 (42.3)  6 (4.4)  22 (16.1)  50 (36.5)  1 (0.7) | 58 (70.7)  6 (7.3)  15 (18.3)  3 (3.7)  0 (0.0) | 0 (0.0)  0 (0.0)  7 (12.7)  47 (85.5)  1 (1.8) |
| **BWH**  **T1, n (%)**  **T2a, n (%)**  **T2b, n (%)**  **T3, n (%)**  **TX, n (%)** | 71 (46.7)  32 (21.1)  39 (25.7)  9 (5.9)  1 (0.6) | 64 (66.0)  17 (17.5)  13 (13.4)  3 (3.1)  0 (0.0) | 7 (12.7)  15 (27.3)  26 (47.3)  6 (10.9)  1 (1.8) |
