# Supplementary material for: C5aR1 Promotes Invasion, Metastasis, and Poor Prognosis in Cutaneous Squamous Cell Carcinoma
Source: Am J Pathol. 2025 Mar 6;195(6):1158–71. doi: 10.1016/j.ajpath.2025.02.004 (PMC12163391; doi:10.1016/j.ajpath.2025.02.004)
Supplement: Supplementary Table S2 [file mmc2.docx]

**Supplemental Table S2**. Tumor and patient characteristics of RDEBSCC tissue samples.

|  | Sex (F/M) | Tumor number | Age at tumor diagnosis (year) | Region | Grade | Metastasis | Death from cSCC |
| --- | --- | --- | --- | --- | --- | --- | --- |
| **Patient 4** | F | **Tumor 8** | 25 | Neck | 2 | Yes | No |
| **Patient 6** | F | **Tumor 1** | 33 | Leg | 2 | Yes | No |
|  |  | **Tumor 2** | 36 | N/A | 3 | Yes | No |
|  |  | **Tumor 3** | 36 | N/A | 3 | Yes | No |
|  |  | **Tumor 4** | 37 | N/A | 3 | No | No |
|  |  | **Tumor 6** | 37 | N/A | 1-3 | No | No |
|  |  | **Tumor 7** | 37 | Arm | 3 | Yes | No |
| **Patient 15** | M | **Tumor 9** | 27 | Hand | 3 | No | N/A |
| **Patient 16** | M | **Tumor 10** | 36 | Leg | 2 | Yes | Yes |
|  |  | **Tumor 11** | 36 | Leg | 3 | Yes | Yes |
| **Patient 17** | F | **Tumor 12** | 57 | Leg | 2 | No | No |

F, female; M, male; N/A, not available
